# Control of Venereal Disease in Australia

**Published:** 1917-11

**Authors:** 


### CONTROL OF VENEREAL DISEASE IN AUSTRALIA.

---

In Western Australia, quite a drastic law has been enacted dealing with venereal disease. It has provisions regulating both the patient and his physician.

**Regulations applying to the Patient**; Everyone suffering from venereal disease must, within three days of his recognition of the fact, place himself under medical care, under penalty of a $100 fine. Such a person must continue under medical care until he receives a certificate of cure, also under like penalty of $100 fine.

If he should change his physician, he must give the name and address of his last medical attendant to his new physician (so that the latter may notify the former of the change).

In the case of a minor (under 16 years of age) afflicted with venereal disease, the parents or guardians are required to secure observance of the act under penalty of $50 fine. Further, under like penalty, they must report to the Commissioner of Health any failure of such a minor to observe the regulations.

When the Commissioner of Health has information that a particular individual is suffering from a venereal disease, he may give written notification to such person requiring him to place himself under medical care or to produce a certificate that he is not afflicted. If the commissioner is not satisfied with such a certificate, he may have the suspect examined by any health officer or by two designated physicians. If the report from such an examination be positive, and it be judged that there be risk of infecting others, the Commissioner may issue a warrant for the arrest and detention, for two weeks, of the person in question, in a hospital, during which time any necessary examination may be effected. If further detention be thought necessary, the governor, on recommendation of the commissioner, may issue a warrant for the arrest of the sufferer and his detention for such time as he thinks fit, and during treatment. Such a detained individual has the right to court appeal, and an examination by two physicians, one of whom he may choose, and who are to certify as to his actual condition. This appeal may not be repeated within six months. Warrants isued under the act authorize the use of force, and require the police, under penalty of $100 fine to lend all required aid.

Further, it is provided that any person who knowingly does, or suffers, any act which infects another with venereal disease, may be fined $250, or be imprisoned, at hard labor, for six months.—Western Medical Review.

